# The cognitive remediation of attention in HIV-associated neurocognitive disorders (HAND): A meta-analysis and systematic review

**DOI:** 10.12688/f1000research.132166.1

**Published:** 2023-09-11

**Authors:** Sizwe Zondo

**Affiliations:** 1 Department of Psychology, Rhodes University, Grahamstown, Eastern Cape, South Africa; 2 Department of Psychology, School of Human and Community Development, University of the Witwatersrand, Braamfontein, Johannesburg, Gauteng, South Africa

**Keywords:** HIV, HAND, Attention Rehabilitation, Neuroplasticity, meta-analysis, meta-regression

## Abstract

**Background:** Despite medical advances in Highly Active Antiretroviral Therapy (HAART), patients living with HIV continue to be at risk for developing HIV-associated neurocognitive disorders (HAND). The optimization of non-HAART interventions, including cognitive rehabilitation therapy (CRT), shows promise in reversing the impact of HAND. No data exist indicating the efficacy of CRT in remediating attention skills following neuroHIV. This paper presents a meta-analysis of randomised and non-randomised controlled trials (RCTs) to remediate attention skills following HIV CRT.

**Methods:** The database search included literature from Google Scholar, ERIC, Cochrane Library, ISI Web of Knowledge, PubMed, PsycINFO, and grey literature published between 2013 and 2022. Inclusion criteria included studies with participants living with HIV who had undergone CRT intervention to remediate attention skills following neuroHIV. Exclusion criteria included case studies, non-human studies, and literature reviews. To assess study quality, including, randomisation, allocation concealment, participant and personnel blinding, the Cochrane Collaboration ratings system was applied.

**Results:** A total of 14 studies met the inclusion criteria (n = 532). There were significant pre- to post-intervention between-group benefits due to CRT in the experimental group relative to control conditions for the remediation of attention skills following HIV acquisition (Hedges g = 0.251, 95% CI = 0.005 to 0.497; p < 0.05). No significant effects (p > 0.05) were demonstrated for subgroup analysis.

**Conclusions:** To the author's knowledge, this is the first meta-analysis that exclusively analyses the remediation of attention skills in the era of HAART and neuroHIV, where all studies included participants diagnosed with HIV. The overall meta-analysis effect indicates the efficacy of CRT in remediating attention skills in HIV and HAND. It is recommended that future cognitive rehabilitation protocols to remediate attention skills should be context and population-specific and that they be supplemented by objective biomarkers indicating the efficacy of the CRT.

**Registration:** Protocols.io (01/03/2023).

## Introduction

The Human Immunodeficiency Virus (HIV) continues to be a significant global pandemic with no known cure. Latest figures from UNAIDS (2021) indicate that by the end of 2021, approximately 38.4 million people were living with the virus, with 1.5 million newly reported cases in 2021 alone. Virologically, HIV is a ribonucleic acid (RNA), single-stranded retrovirus ( Poltronieri *et al.*, 2015) that targets cells in the immune system. Through pathobiology yet to be understood, following transmission to the host, HIV permeates the blood-brain barrier (BBB), where it leads to the differentiation of monocytes into macrophages. This differentiation leads to the infection of cells in the CNS, namely microglia and astrocytes ( Filipowicz *et al.*, 2016; Sillman *et al.*, 2018). In breaching the BBB, HIV is thought to dysregulate the brain’s intrinsic nerve cell architecture, leading to aberrant neural transmission, including excess glutamate levels and decreased dopaminergic transmission ( Elbirt *et al.*, 2015; Nolan & Gaskill, 2019). Markedly, HIV’s viral penetrance and persistence in the CNS is implicated in neuronal apoptosis, leading to a milieu of neurocognitive impairments associated with HIV ( Das *et al.*, 2016; Smail & Brew, 2018).

Neurocognitive impairments resulting from HIV are well-documented and include HIV Dementia ( Hussain *et al.*, 2022; Mattson *et al.*, 2005), HIV-associated neurocognitive disorder (HAND) ( Elbirt *et al.*, 2015; Smail & Brew, 2018), and HIV encephalitis ( Morgello, 2018). Specific to HAND ^1^, although no reliable worldwide estimates exist, the prevalence rates are estimated to range from 25% to 59% of HIV cases ( Bonnet *et al.*, 2013; Elbirt *et al.*, 2015; Kinai *et al.*, 2017). According to updated criteria, HAND is diagnosed if a patient performs more than one standard deviation (SD) below his or her normative mean, on standardized neuropsychological measures, in two or more cognitive domains ( *e.g.*, attention, speed of processing, verbal memory, executive functioning) ( Antinori *et al.*, 2007; Brew, 2018; Chan *et al.*, 2019; Chan & Wong, 2013; Saloner & Cysique, 2017).

### Problem statement

Pharmacological interventions, namely Highly Active Antiretrovirals (HAART), have improved cognitive outcomes in HIV ( Benki-Nugent & Boivin, 2019; Jantarabenjakul *et al.*, 2019). However, to date, their efficacy in treating or reducing the impact of HAND remains variable ( Alford & Vera, 2018; Lanman *et al.*, 2019; Yuan & Kaul, 2019). For example, Underwood *et al.* (2015) found that prolonged treatment with efavirenz (a non-nucleoside reverse transcriptase inhibitor) (NNRTI) and raltegravir (integrase inhibitor) may play a role in HIV-associated cognitive decline and poorer cognitive function in children living with HIV. A cognate study by Hammond *et al.* (2019) found that children in a South African study showed no cognitive gains after receiving efavirenz. The study hypothesised that efavirenz, due to its pharmacokinetic profile (genetically slow metabolites), may be a risk factor for neurotoxicity, leading to the poor neurocognitive outcomes associated with HAND.

Correspondingly, Crowell *et al.* (2015), in their study involving 396 children living with HIV, found an association between early viral suppression and improved neurocognitive outcome; however, they found no association between a high CNS Penetration-Effectiveness score (CPE) ^2^ and neurocognitive improvement in the children. These findings are similar to those reported by Ellis *et al.* (2014), who found no association between high CPE and gains in neurocognition in a longitudinal study conducted among adults living with HIV. Similarly, Puthanakit *et al.* (2013), found no improvement in neurocognitive outcomes amongst 139 Thai and Cambodian children living with HIV after a three-year initiation of cARTs. Other studies ( Das *et al.*, 2016; Iglesias-Ussel & Romerio, 2011; Kumar *et al.*, 2018) indicate that when antiretrovirals (ARVs) act upon the brain HIV viral reservoir, they are indicated to significantly cause neurotoxicity, leading to further neurocognitive and psychiatric impairments ( Alford & Vera, 2018; Das *et al.*, 2016; Lanman *et al.*, 2019; Vázquez-Santiago *et al.*, 2014; Wilmshurst *et al.*, 2018).

### Brain plasticity and cognitive rehabilitation

Given the pharmacological limitations associated with ARVs, including limitations in viral reservoir penetration in the brain and neurotoxicity, studies have begun investigating the efficacy of alternative non-pharmaceutical therapies, namely cognitive rehabilitation therapy (CRT) ^3^, to reverse HAND. The principles of CRT are based on neuroplasticity. Neural brain plasticity posits that the human cortex is malleable and has the inherent capacity to undergo structural and functional change ( Bach-y-Rita, 2003; Wilson *et al.*, n.d.). Within the mammalian cortex, structural and functional change is attendant to continued and repeated exposure to cognitively demanding brain training exercises, purposed to rewire and improve neuronal connectivity, increase blood supply and improve brain function ( Hebb, 1949; Luria, 1948; Merzenich, 2013). Luria (1970), particularly notes that brain plasticity and cognitive training allow for axonal and synaptic connections reintegrating. Accordingly, through dendritic outflow, synaptic connections are thought to stimulate neuronal density and cortical enrichment in near and distant neuronal networks responsible for disparate cortical functions.

Given the promise of positive neuroplasticity to harness neurocortical networks and ameliorate brain function, the extant literature has documented the efficacy of brain training exercises to reduce the risk of cognitive impairment sequent HAND. For example, data indicate improvements in cognitive domains such as executive functions ( Boivin *et al.*, 2016; Frain & Chen, 2018), attention ( Basterfield, & Zondo, 2022; Boivin *et al.*, 2010; Towe *et al.*, 2017), processing speed ( Cody *et al.*, 2020; Vance *et al.*, 2012), and working memory ( Fraser & Cockcroft, 2020; Towe *et al.*, 2017), following intensive brain training exercise in neuroHIV.

Despite the above early promising findings, contradictory findings have been reported. For example, Vance *et al.* (2012) used a computerized CRT program (InSight) to investigate processing speed in adults. Although the experimental group showed significant baseline-to-post-test improvements in speed processing, speed processing deficits are not prevalent in the post-cART era ( Heaton *et al.*, 2011). In another study, Pope *et al.* (2018) found that a computerised program (Posit Science: BrainHQ) could improve abstraction and executive functions, whereas Fazeli *et al.* (2019), reported that the same software enhanced other cognitive domains, such as attention, working memory, and information processing in the experimental group. Moreover, some studies returned insignificant findings ( *e.g.*, Vance *et al.*, 2018; Fazeli *et al.* 2019) when comparing the effect of the cognitive rehabilitation on the experimental group, compared to the control, despite utilizing brain training programs and techniques that have proven to improve cognition in HIV.

Given contradictory findings within the literature, Vance *et al.* (2019), conducted a systematic review of 13 computerised cognitive rehabilitation (CCT) studies investigating the efficacy of cognitive rehabilitation to reverse HAND. The review by Vance *et al.* (2019) found that, for the most part, CRT in HIV was associated with improved cognitive outcomes that translated to improvements in quality of life. Nonetheless, although the systematic review provides summary data on the effect of CRT on working memory, processing speed, and ageing, it does not provide effect size data for each of the reviewed cognitive domains. Most importantly, it does not provide information on the cognitive rehabilitation of attention skills, although deficits in attention are the foremost and common consequence of HIV ( Posada *et al.*, 2012; Wang *et al.*, 2017). Moreover, since the review did not provide effect size data, it did not provide data detailing the effect of moderator variables or subgroup meta-analytic data on HIV cognitive rehabilitation outcomes. Given these limitations, the current study aimed to conduct a meta-analysis investigating the efficacy of CRT in remediating attention skills among people living with HIV. Significantly, the study aimed to (1) provide effect size data detailing pre- and post-intervention improvement in attention due to CRT among people living with HIV. Secondly, the study sought to (2) investigate the effect of moderator variables by conducting subgroup analyses of the effect size (if significant). Lastly, the study aimed to provide clinical suggestions for implementing HIV CRT interventions in low-to medium-income countries with a high number of HIV cases.

## Methods

This study was registered on Protocols.io ( dx.doi.org/10.17504/protocols.io.5jyl8jqm7g2w/v1; 01/03/2023). Although the study was not registered in PROSPERO the review protocol can be found as *Extended data* ( Zondo, 2023). The study followed the Preferred Reporting Items for Systematic Reviews and Meta-analyses (PRISMA) reporting guidelines for meta-analyses ( Moher *et al.*, 2009).

### Search strategy

The units of analysis were chosen from published literature containing medical subject headings (MeSH) and text words related to: ‘HIV and attention rehabilitation’ or ‘HIV and cognitive rehabilitation’, ‘HIV and/or attention’, ‘HIV and attention remediation’, ‘HIV and executive attention remediation’. These were combined with terms related to outcome research such as: ‘effect’, ‘efficacy’ ‘evaluation’, or ‘outcome’. To identify relevant studies, journal articles, books, dissertations, and electronic databases were searched. Electronic database searches included Google Scholar (RRID:SCR_008878), ERIC (RRID:SCR_007644), Cochrane Library (RRID:SCR_013000), ISI Web of Knowledge, PubMed (RRID:SCR_004846), and PsycINFO (RRID:SCR_014799). Grey literature and unpublished papers were searched based on indices of conference proceedings and dissertation abstracts to minimise publication bias ( Higgins & Green, 2008). A complete breakdown and description of the search strategies are available as *Extended data* ( Zondo, 2023).

### Inclusion criteria

The inclusion criteria for studies included in the meta-analysis were included based on the PICOS criteria. The population and condition of interest were patients diagnosed with HIV, receiving cognitive rehabilitation to remediate attention. Participants in the experimental group should have undergone an intervention to remediate attention skills following neuroHIV. Studies should have included a comparison between the experimental group and at least one control group (passive control group, or active control group). Studies should also have reported outcome data in the form of pre-and post-intervention attention scores using validated neuropsychological measures of attention ^4^. Lastly, studies were included if they used random and non-random control trial study designs. In addition to the PICOS criteria, studies should have reported sample sizes to enable effect size weighting ( Borenstein *et al.*, 2021).

**Figure 1.**
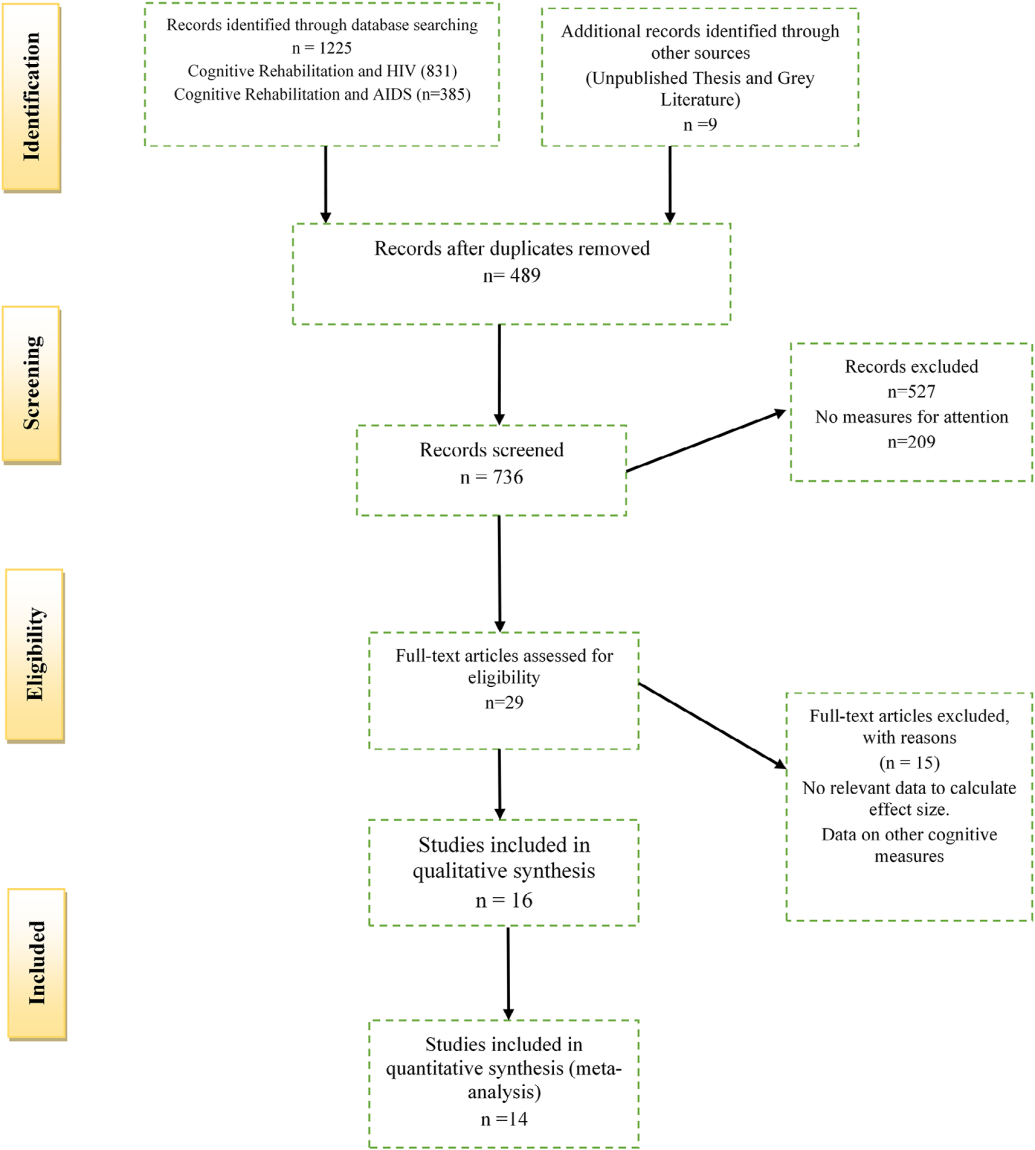
PRISMA flow diagram depicting the selection of studies for the meta-analysis.

The above inclusion criteria were combined with the 27-item PRISMA checklist to produce a four-phase PRISMA flow diagram as suggested by Moher *et al.* (2009). The complete PICOS criteria accompanied by the PRISMA checklist can be found as *Extended data* ( Zondo, 2023). The PRISMA flow diagram is indicated below in Figure 1. All duplicate studies for the meta-analysis were removed using Mendeley Data (RRID:SCR_002750) Software, Version 1. The author (S.Z) and two researcher assistants (K.S and T.C), independently assessed the titles and abstracts retrieved from literature searches for relevance. After the initial assessment, the same reviewers determined the eligibility of all full-text relevant for the meta-analysis. Any disagreements (three articles) were resolved by a third research assistant (N.M) with expertise in meta-analytic research. Relevant but excluded studies from the reviewed literature are indicated in the *Extended data* ( Zondo, 2023).

### Data extraction

Data extraction was done by the author and cross-checked (by K.S. and T. C.) using an Excel spreadsheet (Microsoft Corporation) (RRID:SCR_016137). The relevant summary statistics to investigate attention outcomes due to cognitive rehabilitation included individual data points linked to: (a) The number of participants in each of the groups, (b) relevant statistical data such as means, and standard deviations, for both the experimental and control groups (active or passive control) on the attention measures, pre and post the intervention ( Field & Gillett, 2010; Lee, 2018).

The extraction of attention outcomes (dependent variable) for each of the studies used in the meta-analysis are available as *Extended data* ( Zondo, 2023). Other key moderator data extracted from each of the studies included in the meta-analysis included data related to: (a) the duration of the cognitive training exercises (<10 sessions; 10 sessions; >10 sessions in each study; (b) the type of cognitive training received (computerised; pencil and paper, mixed); (c) the setting of the cognitive training (individualised training; group intervention); (d) the type of research design employed (random control trial *vs.* non-random control); (e) the socio-economic setting of the study (High *vs.* Low); (f) the data quality of the study (Low, Medium, High); (g) the type of sample (paediatric HIV *vs.* geriatric HIV); (h) the type of control group utilised (active, passive, both) and (g) whether participants were blind or aware to their condition (experimental or control). The above variables were included in the subgroup meta-analysis to investigate the influence of pertinent moderator variables on the overall effect size of the cognitive rehabilitation.

### Data analysis

The meta-analysis was performed using Stata 17 (StataCorp. 2021. Stata Statistical Software: Release 17. College Station, TX. StataCorp, LLC) (RRID:SCR_012763) (free alternative, RStudio). The overall effect size was determined by measuring attention scores pre-and-post the CRT to investigate the effectiveness of the attention intervention. Given the heterogeneity across all studies, the random effect model was used to estimate the pooled effect ( Borenstein *et al.*, 2009). Effect sizes were calculated based on the standardised mean difference (SMD) sizes for the individual studies, weighted according to the relevant sample size. Briefly, when using SMD, effect is calculated as the mean change in pre-intervention scores compared to post-intervention scores in the intervention group minus the mean change from pre-intervention to post-intervention scores in the control group, divided by the combined pre-intervention standard deviation scores ( Borenstein *et al.*, 2009; Field & Gillett, 2010).

Following suggestions from Borenstein *et al.* (2021), the inverse variance method was used to interpolate the SMDs of each study. As suggested by Borenstein *et al.* (2021), since the SMD model also corrects for the use of different outcome measures ( *i.e.*, different neuropsychological outcomes used to assess attention), it, however, fails to account for differences in the direction of the participants’ behavioural performance (neuropsychological performance) within the various outcome measures used in the meta-analysis (neuropsychological assessments). Stated differently in certain assessments, an increase in mean scores may represent a decline in disease severity, whereas, in some neuropsychological assessments, a decrease in mean scores represents a decline in disease severity ( Borenstein *et al.*, 2009). To correct these differences, mean scores from studies in which a decrease in mean scores represents a decline in disease severity were multiplied by -1, ensuring conformity of direction for all the scales used in the meta-analysis calculation ( Borenstein *et al.*, 2009). Based on the above recommendations, the mean scores for both the control and experimental group from the following studies Fazeli *et al.* (2019), Zondo and Mulder (2015) were corrected by multiplication of -1.

The H ^2^ and I ^2^ statistic (based on the *Q* statistic) was used to assess the proportional significance of heterogeneity ( Borenstein *et al.*, 2009). The author tested for publication bias using a funnel plot, which is a type of scatterplot with treatment effect size (Cohen’s D) plotted on the x-axis, and the standard error (variance) plotted on the y-axis ( Borenstein *et al.*, 2009). Statistical significance for all analyses was set at a threshold of p < 0.05.

## Results

### Search results

Since the inclusion criteria of the studies are detailed in the methods section, a PRISMA flow chart indicating the decision process and study selection is described in Figure 1. In summary, the study included a total of 1,225 records; after removing duplicates (489), 736 records were screened. A further 527 records were excluded based on title searches, abstracts, and methodological considerations ( *i.e.*, case studies and qualitative designs). A total of 15 studies were deemed relevant but were excluded from the analysis due to reasons provided as *Extended data* ( Zondo, 2023). In total, 29 studies were assessed for eligibility with the final selection including 11 randomised control studies ( Basterfield, & Zondo, 2022; Boivin *et al.*, 2010; Casaletto *et al.*, 2016; Cody *et al.*, 2020; Fazeli *et al.*, 2019; Frain & Chen, 2018; Fraser & Cockcroft, 2020; Livelli *et al.*, 2015; Ownby & Acevedo, 2016; Pope *et al.*, 2018; Vance *et al.*, 2012) and three non-randomised studies ( Cody *et al.*, 2015; Ezeamama *et al.*, 2020; Zondo & Mulder, 2015).

### Study characteristics

The characteristics of all studies included in the meta-analysis are detailed in Table 1. The analysis included data from South Africa ( Basterfield, & Zondo, 2022; Fraser & Cockcroft, 2020; Zondo & Mulder, 2015), Uganda ( Boivin *et al.*, 2010; Ezeamama *et al.*, 2020), Italy ( Livelli *et al.*, 2015), and the US ( Casaletto *et al.*, 2016; Cody *et al.*, 2015, 2020; Fazeli *et al.*, 2019; Frain & Chen, 2018; Ownby & Acevedo, 2016; Pope *et al.*, 2018; Vance *et al.*, 2012). In total, the study comprised 532 participants (255 participants in the intervention group and 277 participants in the control group).

**Table 1.** Study and participant characteristics. CRT, cognitive rehabilitation therapy; HIV, Human Immunodeficiency Virus; HAART, Highly Active Antiretroviral Therapy; ARV, antiretroviral; HAND, HIV-associated neurocognitive disorders.

Study	Sample								Attention remediation group					
	Sample size			Age mean in years		Female (n)		Overall description of participants	Training received	Number of sessions ^b^	Sessions per week	Follow up duration	Type of attention training	Control condition
	Total	CRT	Control	CRT	Control	CRT	Control							
Basterfield & Zondo (2022), SA	5	3	2	11.27	11.23	2 (66)	1(50)	Children ^a^ (aged 10-15 yrs.) with HIV receiving HAART.	BrainWaveR	8	3	None	Selective	Microsoft Word Exercises
Boivin *et al.* (2010), Uganda	60	32	28	10.34	9.36	21(65.6)	15(53.6)	Children (aged 6-16 yrs.) with perinatal transmission of HIV on HAART.	Captain’s Log	10	2	None	Simple	Non-Active Cognitive Training
Casaletto *et al.* (2016), USA	90	30	30 x 2	50.1	47.8	3 (10)	7 (23)	Adults (47-51 yrs.) with HIV and Substance Use Disorder and Dysexecutive Syndrome.	Goal Management & Metacognition Training	1	N/A	None	Divided	Paper Origami Exercises
Cody *et al.* (2015), USA	20	13	24	50.22	52.9	4 (25)	(7) 29	Adults (47-51 yrs.) with asymptomatic HIV.	PositScience	5	N/A	1-2 Months	Selective& Divided	No Contact
Cody *et al.* (2020), (USA)	33	17	16	58.82	62.12	7 (33.3)	5 (31)	Adults (>55 yrs.) with and without HIV, and no Hx of brain trauma.	PositScience Transcranial Deep Stimulation (tDCS)	10	2	N/A	Selective& Divided	Sham tDCS

Study	Sample								Attention remediation group					
	Sample size			Age, Mean Years		Female, sex, %		Overall description of participants	Training received	Number of sessions	Sessions per week	Follow up duration	Type of attention training	Control condition
	Total	CRT	Control	CRT	Control	CRT	Control							
Ezeamama *et al.* (2020), Uganda	81	41	49	59.7	60.0	24 (59)	25 (50)	Elderly adults (>50 yrs.) with HIV and without other mental or health disorders.	Captain’s Log	10	2	5 Weeks	Simple	Standard of Care (SOC)
Fazeli *et al.* (2019), USA	33	17	16	56	55.63	6 (35)	5 (31)	Adults (>50 yrs.) with HIV, and without a history of brain trauma, and/or mental health disorders.	PositScience BrainHQ	10	N/A	4 Weeks	Selective & Divided	Sham tDCS
Frain & Chen (2018), USA	22	10	12	58	54	0 (0)	3 (25)	Adults (>50 yrs.) with HIV, on ARVs.	PositScience BrainHQ	N/A	3	2 and 4 Months	Visual Attention	Nonactive Cognitive Training
Fraser & Cockcroft (2020), SA	63	31	32	12.0	12.41	16 (52)	16 (50)	Adolescents (aged 10-15 yrs.) diagnosed with Clad C HIV on HAART.	Jungle- Memory Computer Exercises	32	4	6 months	Selective & Sustained	Microsoft Paint Exercises
Livelli *et al.* (2015), Italy	32	16	16	47.5	50.0	5 (31)	3 (19)	Adults (>50 yrs.) with HAND receiving care (Amedeo di Savola Hospital).	Mixed: Pencil and Paper & Computer Exercises	36	N/A	6 months	Selective Divided Sustained Divided	Standard Care (SOC)
Ownby & Acevedo (2016), USA	11	5	6	50.3	52.8	0	2 (40)	Adults (>51 yrs.) with HIV, and self-reported cognitive impairment in at least 2 cognitive domains.	GT Racing2 Game & tDCS	6	N/A	N/A	Simple Attention	Sham tDCS
Pope *et al.* (2018), USA	30	15	15	55.3	53.7	5 (33)	6 (40)	Adults (>50 yrs.) with HIV, and without a history of brain trauma, and/or mental health disorders.	PositScience	10	4	N/A	Divided & Selective	Sham tDCS
Vance *et al.* (2012), USA	46	22	24	50.1	52.9	5 (23)	(7) 29	Adults (47-51 yrs.) with asymptomatic HIV.	PositScience	10	N/A	N/A	Visual Attention	No Contact
Zondo & Mulder (2015), SA	6	3	3	11	11	0	0	Children (11-year-old) with HIV receiving HAART.	BrainWaveR	8	2	N/A	Selective	No Contact

^a^ The definition ‘child’, is based on age guidelines from the UN Convention of the Rights of the Child that describe the child/paediatric age as ranging from birth to adolescence (16-19 years) (UN Convention Assembly, 1989).

^b^ In most studies ( *e.g.*, Fraser & Cockcroft, 2020), a session lasted for a period of 30-45 mins.

As indicated in Table 1, participant ages for adults ranged from a mean of 47.5 years ( Livelli *et al.*, 2015) to 59.7 years ( Ezeamama *et al.*, 2020) in the intervention group; and 50.0 years ( Livelli *et al.*, 2015) to 62.12 years ( Cody *et al.*, 2020) in the control group. Participant ages in the paediatric HIV groups ranged from a mean of 10.34 years ( Boivin *et al.*, 2010) to 12.0 years ( Fraser & Cockcroft, 2020) in the intervention group and 9.36 years ( Boivin *et al.*, 2010) to 12.41 years ( Fraser & Cockcroft, 2020) in the control group. The proportion of female participants ranged from 0% ( Frain & Chen, 2018) to 66% ( Boivin *et al.*, 2010) in the intervention group and 0% ( Zondo & Mulder, 2015) to 90% ( Boivin *et al.*, 2010) in the control group.

The ‘types’ of attention intervention implemented varied extensively and included selective attention training ( Basterfield, & Zondo, 2022), divided attention training ( Casaletto *et al.*, 2016), selective and divided attention training ( Fazeli *et al.*, 2019), selective, divided, and sustained attention training ( Fraser & Cockcroft, 2020; Livelli *et al.*, 2015), visual attention ( Frain & Chen, 2018; Vance *et al.*, 2012), and simple attention training ( Ezeamama *et al.*, 2020; Ownby & Acevedo, 2016). Nine of the studies ( Boivin *et al.*, 2010; Casaletto *et al.*, 2016; Ezeamama *et al.*, 2020; Fazeli *et al.*, 2019; Frain & Chen, 2018; Livelli *et al.*, 2015; Ownby & Acevedo, 2016; Pope *et al.*, 2018; Vance *et al.*, 2012) reported participant biomarker data, in the form of CD4+ T-cell count, pre-and post-the intervention. CD4+ T-cell counts in the treatment group ranged from 552 cells/μL ( Casaletto *et al.*, 2016) to 833 cells/μL ( Fazeli *et al.*, 2019). None of the studies reported biomarker data in the form of neuroimaging data ( *i.e.*, MRI, EEG, fNIRS) detailing the effects of the intervention from baseline to post-intervention changes.

The total number of rehabilitation sessions ranged from 1 ( Casaletto *et al.*, 2016) to 36 sessions ( Livelli *et al.*, 2015). There was much variability within the studies regarding the duration of each rehabilitation session, with some sessions, ranging from 10-15 minutes per session ( Casaletto *et al.*, 2016) to others ranging from 30-45 minutes per session ( Fraser & Cockcroft, 2020). Training frequency within studies ranged from two sessions per week ( Boivin *et al.*, 2010) to four sessions per week ( Fraser & Cockcroft, 2020; Pope *et al.*, 2018). For most studies, the control conditions were divided into one of two control types: either active controls (six studies: Basterfield & Zondo, 2022; Casaletto *et al.*, 2016; Cody *et al.*, 2020; Ezeamama *et al.*, 2020; Fazeli *et al.*, 2019; Fraser & Cockcroft, 2020; Livelli *et al.*, 2015), or passive controls (five studies: Boivin *et al.*, 2010; Cody *et al.*, 2015; Frain & Chen, 2018; Vance *et al.*, 2012; Zondo & Mulder, 2015). None of the reviewed studies included *both* active and passive controls, to assess the impact of the active ingredient (cognitive rehabilitation), compared to the influence of a sham activity (active control) and/or passive interaction (passive control).

Overall, 10 of the studies implemented computerised cognitive rehabilitation (CCT) protocols ( Boivin *et al.*, 2010; Cody *et al.*, 2015, 2020; Ezeamama *et al.*, 2020; Fazeli *et al.*, 2019; Frain & Chen, 2018; Fraser & Cockcroft, 2020; Ownby & Acevedo, 2016; Pope *et al.*, 2018; Vance *et al.*, 2012, 2018); two utilised pencil and paper protocols ( Basterfield & Zondo, 2022; Zondo & Mulder, 2015), whereas one study employed a mixture of computer and paper-pencil protocols ( Livelli *et al.*, 2015), whilst another used a mixture of Goal Management and individualised metacognition training ( Casaletto *et al.*, 2016). Of the 10 studies that implemented computerised CRT, two ( Cody *et al.*, 2020; Fazeli *et al.*, 2019) coupled computerised training with transcranial deep brain stimulation (tDCS). Moreover, of the studies that utilised computerised interventions, six used the PositScience-BrainHQ system ( Cody *et al.*, 2015, 2020; Fazeli *et al.*, 2019; Frain & Chen, 2018; Pope *et al.*, 2018; Vance *et al.*, 2012), whereas two employed the Captain’s Log ( Boivin *et al.*, 2010; Ezeamama *et al.*, 2020), which trains multiple cognitive domains including working memory and executive skills, and one made use of Jungle-Memory ( Fraser & Cockcroft, 2020), which trains working memory.

### Meta-analysis of attention

**Figure 2.**
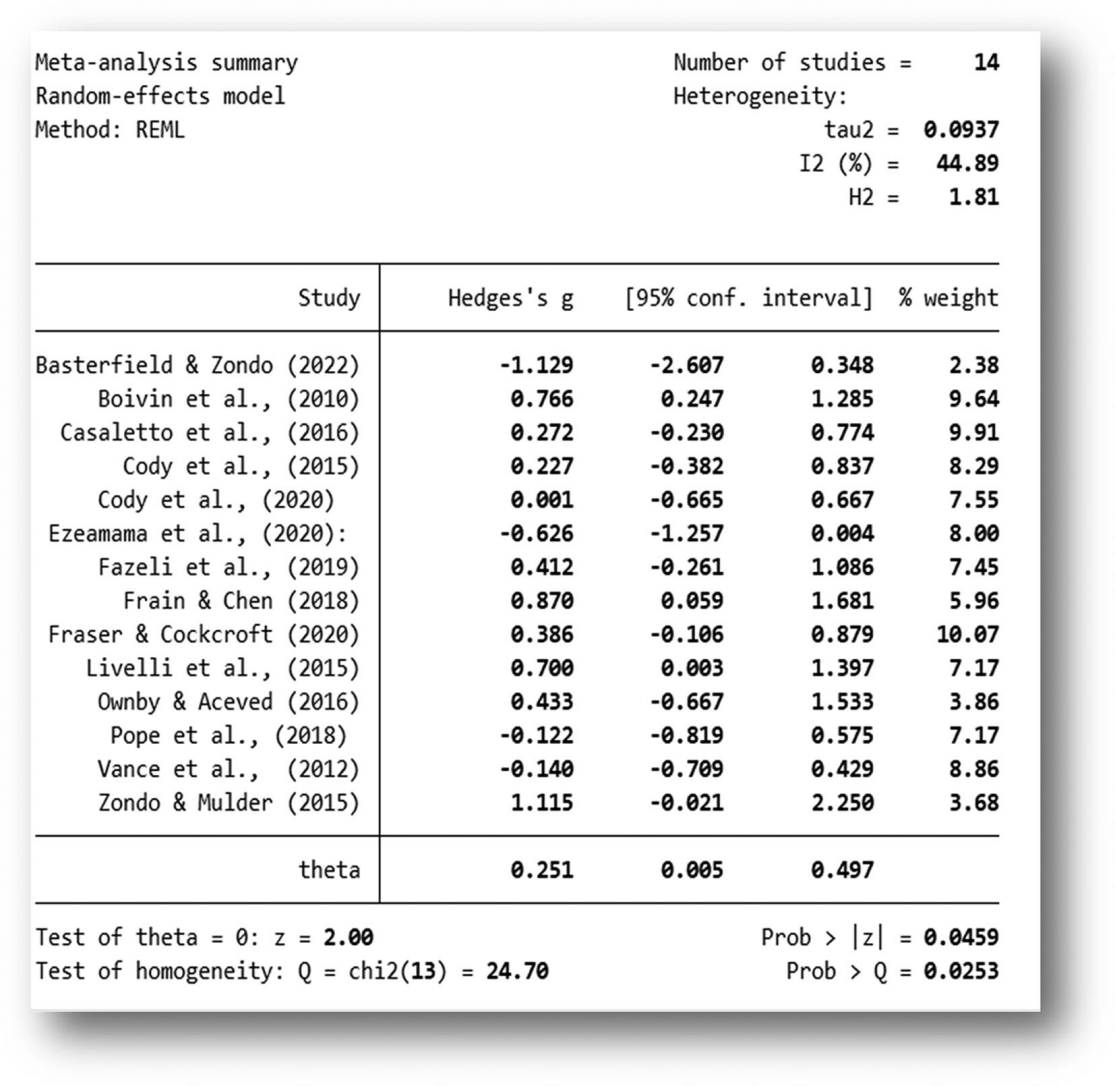
Mean difference. *Note:* The overall standardized mean difference was estimated using the restricted maximum-likelihood estimate (REML).

The analysis was carried out using the standardized mean difference as the outcome effect measure. As indicated in Figure 2, a random-effects model was fitted to the data. The amount of heterogeneity (tau ^2^) was estimated using the restricted maximum-likelihood estimator ( Viechtbauer, 2010). In addition to the estimate of tau ^2^, the Q-test for heterogeneity and the I ^2^ statistic was conducted on a total of 14 studies included in the analysis. The observed standardized mean differences ranged from -1.129 to 1.115, with the majority of estimates being positive (71%). The estimated average standardized mean difference based on the random-effects model was Hedges, *g* = 0.251 (95% CI: 0.005 to 0.4977).

**Figure 3.**
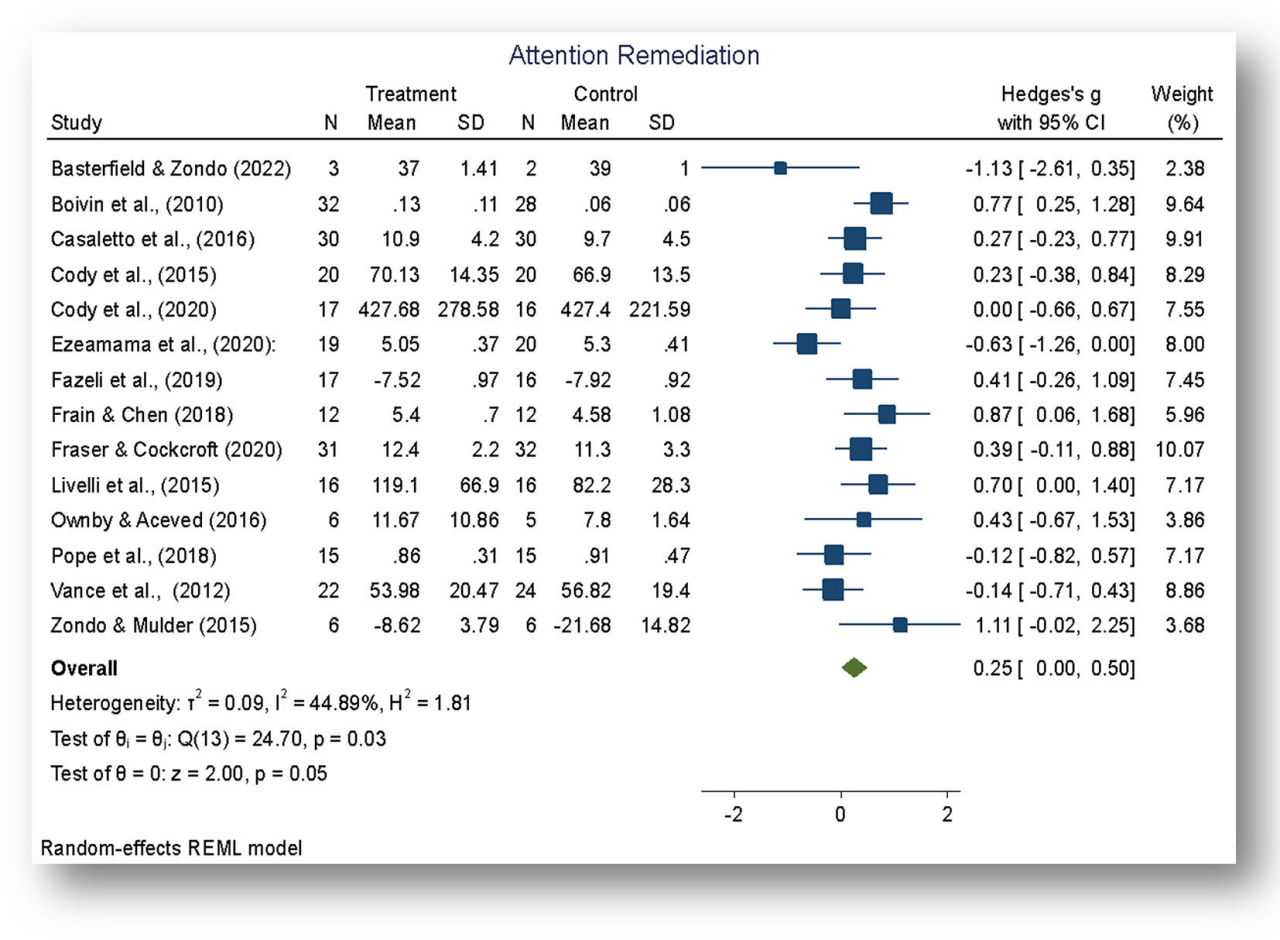
Forest plot. *Note:* The overall effect of the cognitive rehabilitation to remediate attention skills following neuroHIV was significant, z = 2.00, p < 0.05.

The average outcome for attention rehabilitation differed significantly from zero (z = 2.00, p = 0.045). According to the Q-test, the true outcome of the effect size appears to be heterogeneous (Q (13) = 24.70, p = 0.025, tau ^2^ = 0.097, I ^2^ = 44.89%). A 95% prediction interval for the true outcomes of the intervention ranged from - 0.3901 to 0.8579. Although the average outcome for the rehabilitation was estimated to be positive (Hedges g = 0.251, p = 0.045), the data indicate that in some studies the true outcome of the rehabilitation may in fact be negative. Further examination of the studentized residuals revealed that none of the studies had a value larger than ± 2.9137. Hence, there was no indication of outliers in the context of this model. As further indicated by the Forest Plot ( Figure 3), there was greater variability in the 95% CIs in studies with smaller sample sizes and larger weights in studies with post-intervention follow-ups and larger sample sizes.

### Subgroup analysis

Subgroup analyses are presented in Table 2. These were conducted to investigate the effect of key moderator variables, namely (a) the duration of the intervention (<10 sessions, 10 sessions, >10 sessions), (b) the type of rehabilitation (computerized, pencil and paper, mixed), (c) the setting of the rehabilitation (individualized or group), (d) the type of research design employed (randomized, non-randomised), (e) data quality rating (Low median, high), (f) the population of the study (paediatric HIV or geriatric HIV), (g) the type of control group in the study (active control, passive control), and (h) the blinding of subjects (aware or blind). No significant subgroup differences were found on any of the moderator variables. Further meta-regression analysis could not be conducted on the data, despite the significant outcomes of the cognitive rehabilitation (Hedges *g* = 0.25, p < 0.05).

**Table 2.** Subgroup analysis. HIV, Human Immunodeficiency Virus.

Table moderator effects (Post test)					
Outcome measure	Criteria	Subgroup (study)	n	Hedges’ g (95% CI) ^ns^	Test of subgroup differences ^a^
Remediation of Attention	Duration	≤10 session	6	0.47 (0.15–0.78)	Q=2.43, df=2 (p=0.31)
		10 Sessions	6	0.06 (-0.34–0.47)	
		≥10 Session	3	0.23 (-0.53–0.99)	
	Type of Rehabilitation	Computerised	12	0.24 (-0.01–0.49)	Q=1.52, df=2, (p=0.46)
		Pencil and Paper	2	0.04 (-2.15–2.24)	
		Mixed	1	0.27 (0.03–1.40)	
	Setting	Individualised	5	0.38 (-0.20–0.96)	Q=0.17, df=1, (p=0.68)
		Group Rehabilitation	10	0.25 (-0.04–0.53)	
	Research Design	Randomization	12	0.33 (0.10–0.56)	Q=0.14, df=1, (p=0.71)
		None Randomized	3	0.15 (-0.77–1.07)	
	Socio Economic Setting	High	10	0.26 (0.05–0.48)	Q=0.06, df=1, (p=0.81)
		Low	5	0.17 (-0.55–0.89)	
	Data Quality	Low	4	0.37 (-0.5–1.24)	Q=0.73, df=2, (p=0.68)
		Medium	4	0.07 (0.59–0.73)	
		High	7	0.37 (0.15–0.60)	
	Population	Paediatric	4	0.48 (-0.04–1.00)	Q=0.94, df=1, (p=0.33)
		Geriatric HIV	11	0.19 (-0.07–0.46)	
	Type of Control	Active	8	0.11 (-0.22–0.44)	Q=2.15, df=2, (p=0.34)
		Passive	4	0.51 (-0.02–1.05)	
		No control	3	0.43 (-0.06–0.91)	
	Blinding	Aware	11	0.17 (-0.13–0.46)	Q=3.27, df=2, (p=0.19)
		Blind	3	0.57 (0.10–1.04)	

ns: All the subgroup analysis were not significant at the 0.05 level of significance.

^a^ Heterogeneity measures for the each of the group analysis.

### Study quality and risk of bias

Study quality assessment ratings were conducted on all studies based on criteria established by the Cochrane Collaboration ( Cumpston *et al.*, 2019; Higgins & Green, 2008). The criteria for quality assessment were as follows: (1) adequate randomization concealment of participants to either the treatment or control group (by the Primary Investigator (s); (2) Blinding of participants to either the treatment or control condition(s) ^5^; (3) Baseline comparability, detailing whether the experimental and control group(s) were comparable on all outcome measures from baseline to post-intervention; (4) Power analysis: Did the study have adequate power and/or at least 15 participants per group for comparative analysis (experimental *vs.* control)? (5) Completeness of follow-up data: Was there adequate follow-up of at least three months post the intervention, with clear attrition analysis of data? (6) Handling of missing data: Were multiple imputation analysis and/or maximum likelihood analysis (or other advanced statistical techniques) applied to account for missing data and high attrition rates? Each of the above criteria was rated as 0 (the study does not meet criterion) or 1 (the study meets criterion). In summary, all studies were rated as Low (score 1 or 2), Medium (score 3 or 4), and High (Score 5 or 6) following suggested guidelines by Cochrane collaboration.

**Figure 4.**
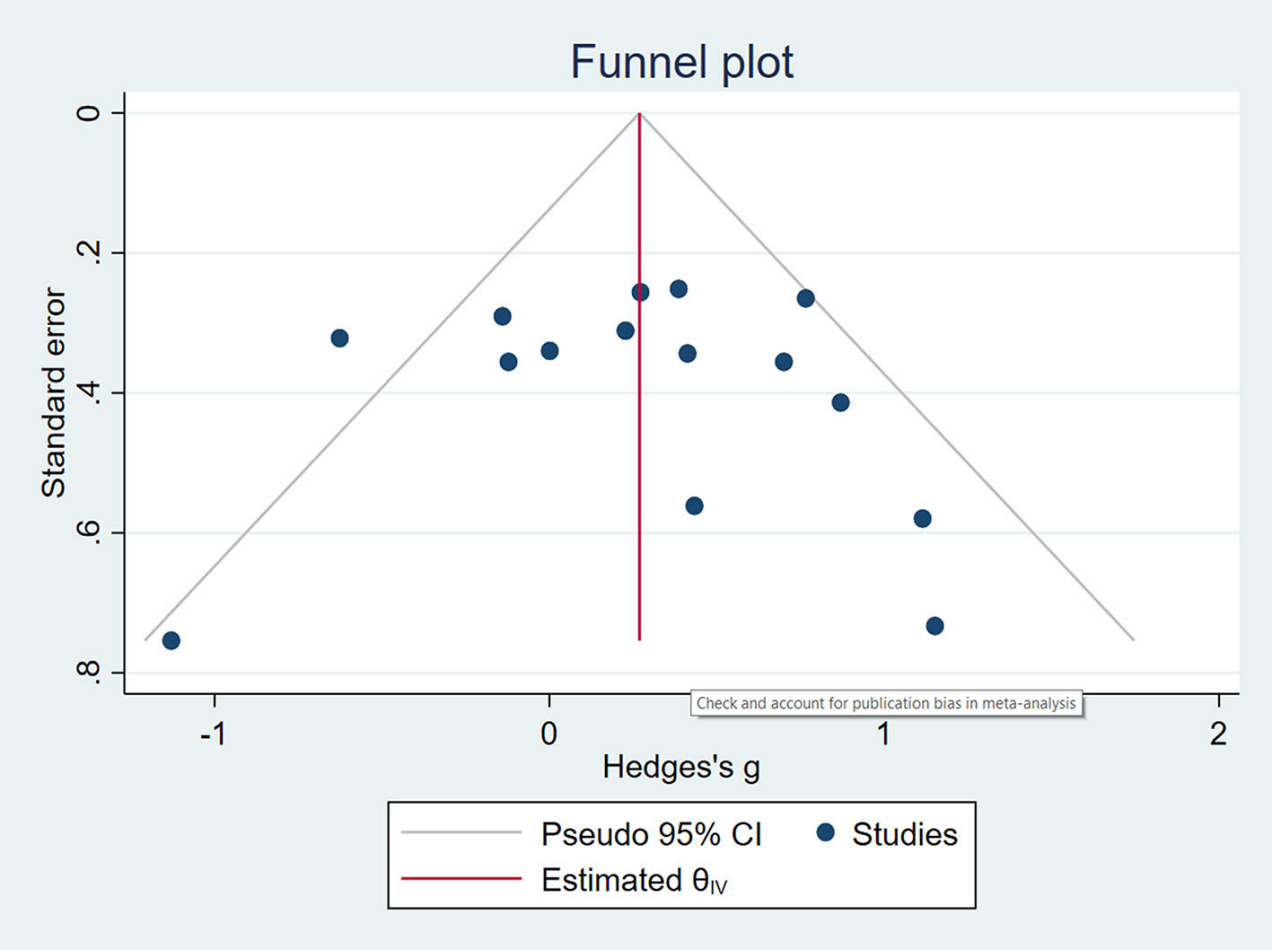
Publication bias. *Note*: According to the funnel plot, only one study could indicate publication bias.

Based on the above criteria, five studies ( Boivin *et al.*, 2010; Casaletto *et al.*, 2016; Fazeli *et al.*, 2019; Fraser & Cockcroft, 2020; Livelli *et al.*, 2015) had high-quality evidence. Six studies ( Cody *et al.*, 2020; Ezeamama *et al.*, 2020; Frain & Chen, 2018; Ownby & Acevedo, 2016; Pope *et al.*, 2018; Vance *et al.*, 2012) had moderate-quality evidence, and three studies ( Basterfield & Zondo, 2022; Cody *et al.*, 2015; Zondo & Mulder, 2015) had low-quality evidence. Summary data for the quality assessment of each study are available as *Extended Data* ( Zondo, 2023). The studies with low-quality ratings primarily presented with small sample sizes and had no follow-ups of at least three months post-intervention and presented large 95% CIs ( *e.g.*, Basterfield & Zondo, 2022; Zondo & Mulder, 2015). Expectedly, the studies with high-quality ratings implemented randomisation, including blinding research participants to group allocation, and had adequate follow-up assessments of at least three months post-intervention. Further indication for study quality and publication bias based on Cook's distances indicated that one study ( Ezeamama *et al.*, 2020) could be overly influential on the meta-analysis effect. Nonetheless, in terms of publication bias, neither the rank correlation nor the regression test indicated any funnel plot asymmetry (p = 0.6265 and p = 0.3459, respectively), but one study was identified with publication bias as indicated in Figure 4.

## Discussion

### Main findings

To the author’s knowledge, this is the first meta-analysis to specifically investigate the efficacy of cognitive rehabilitation therapy as it pertains to brain training to remediate attention in neuroHIV. The nascent brain plasticity literature indicates intrinsic functional connectivity, particularly within the frontoparietal brain network, following attention and working memory cognitive rehabilitation training ( Astle *et al.*, 2015; Marek & Dosenbach, 2022; Spreng *et al.*, 2010, 2013). Based on the scalability of attention and working memory cognitive training, the current meta-analysis found a small but significant effect (Hedges *g =* 0.25, p < 0.05) for the cognitive rehabilitation of attention following HIV acquisition, pre- and post-the rehabilitation.

Findings from this meta-analysis are consistent with previous studies indicating the efficacy of cognitive rehabilitation to train and remediate attention skills in patients with attention deficit-hyperactivity disorder (ADHD) ( Bikic *et al.*, 2018; Wexler *et al.*, 2021), autism ( Spaniol *et al.*, 2018), as well as children ( Astle *et al.*, 2015; Schrieff-Elson *et al.*, 2017), and adults ( Rosenbaum *et al.*, 2018; Spreng *et al.*, 2010) without ADHD. Nonetheless, despite the significant findings indicated in the overall meta-analysis regarding the efficacy of attention remediation in HIV, insignificant effects were noted on key sub-group moderator effects contrary to expectation.

Noteworthy research ( *e.g.*, Azouvi, 2015; Shawn Green *et al.*, 2019; Shoulson *et al.*, 2012; Simons *et al.*, 2016) indicate that multiple moderator effects, including methodological standards, such as the type(s) of the control group employed, randomization and blinding of subjects, and other contextual factors (the social environment of the rehabilitation), including the setting of intervention (group *vs.* individual rehabilitation) ( das Nair *et al.*, 2016; Lincoln *et al.*, 2020), influence cognitive rehabilitation outcomes in brain training protocols.

Within the current meta-analysis, presumably, the null effect observed at the subgroup level may be a result of (a) the limited number of studies and the small number of participants in some of the studies, which might have resulted in insignificant findings being noted at the subgroup level. Secondary to the above, given the variegated nature of ‘attention types’ remediated in the various studies ( *i.e.*, sustained, selective, divided, and simple attention) may have led to a lack of uniformity in the analysis, despite controlling for, and applying the standardised mean difference (SMD) as suggested in the meta-analysis literature ( Borenstein *et al.*, 2009).

### Study limitations

There continues to be a dearth of research investigating the efficacy of cognitive rehabilitation therapy in neuroHIV, and as such, there were limitations on the number of studies that could be included in the analysis. It is thus possible that the strict inclusion criteria (data points reporting ‘attention’ rehabilitation), permitted a weak interpretation of the treatment effect of the cognitive rehabilitation in the current study. The above observation is particularly significant, given that most studies investigating cognitive rehabilitation in the current era of neuroHIV have tended to focus on re-establishing cognitive functions related to ‘executive functions’, ‘working memory’, ‘processing speed’, and ‘aging’ ( Vance *et al.*, 2019). As such, in some studies ( *e.g.*, Ezeamama *et al.*, 2020; Fraser & Cockcroft, 2020), the cognitive rehabilitation of attention was a secondary consideration to the study's main objectives ( *e.g.*, to remediate executive functions’ or working memory in neuroHIV).

Moreover, considering the somewhat limited evidence base for the cognitive rehabilitation of attention in neuroHIV, the current study included data from both paediatric and geriatric HIV populations. Resultantly, the age-heterogeneous nature of participants may have affected the results as indicated by high 95% confidence intervals of some paediatric studies ( *e.g.*, Basterfield & Zondo, 2022). To this end, although research indicates that younger brains have a greater susceptibility to cognitive training compared to adult brains ( Luria, 1970), conducting a meta-analysis on a composite ‘group’ (paediatric and geriatric) at different levels of brain plasticity may have resulted in different magnitudes of observed effects within the analysis. Additionally, as noted within the brain science literature ( *e.g.*, Boot *et al.*, 2013; Simons *et al.*, 2016), there continues to be a preponderance of cognitive rehabilitation studies to compare the effects of the cognitive intervention to passive, inactive controls. As noted by Boot *et al.* (2013), although this approach is expedient, it limits the vigorous interpretation of the ‘active ingredient’ within the rehabilitation and fails to control for the placebo effect that may be present within the intervention group. Significantly, the inclusion of ‘active control groups’, in brain research serves the dual purpose of mitigating the placebo effect and subsequently aids in matching perceived expectations of the cognitive rehabilitation within the treatment group. Unfortunately, none of the studies included in the meta-analysis had active control groups in order to match ‘the active ingredient’ within the intervention group in order to establish causal inference and treatment potency resulting from the intervention, resulting in a major limitation in the current meta-analysis.

## Conclusions

Based on the current body of literature, coupled with findings from the current meta-analysis, there appears to be reasonable evidence to suggest the efficacy of cognitive rehabilitation to remediate attention dysfunction in neuroHIV. Nonetheless, more studies are required to confirm these nascent findings, especially in contexts such as Sub-Saharan Africa, where the high incidence of HIV/AIDS continues to be a significant risk factor for HAND. Following a review of the HIV literature, the suggestions below should be considered when designing cognitive rehabilitation protocols in Sub-Saharan Africa (SSA) and other low-to medium-income countries with a high number of HIV cases.

### Clinical implications and recommendations for future research

Although the reviewed literature indicates that random control trial studies incorporating individualized interventions coupled with intensive intervention protocols ( *i.e.*, 30 sessions of 30-45 minutes per session) ( *e.g.*, Frain & Chen, 2018; Fraser & Cockcroft, 2020) generate larger effect sizes, the nature of brain training intervention research tends to be taxing, time-consuming, and resource heavy, and tends to be associated with high attrition rates ( Ballieux *et al.*, 2016; Salkind, 2015; Schrieff-Elson *et al.*, 2017).

It is thus suggested that future studies could benefit from adopting the Single Case Experimental Designs (SCED) to study the efficacy of CRT as it pertains to neuroHIV in SSA. The benefits of the SCED approach include in-depth rehabilitation sessions (30–45 min) with a limited number of participants (6-8) for prolonged periods of intervention. Their adoption could help mitigate high attrition rates often observed with paediatric intervention research, further improving the internal validity of neuroHIV rehabilitation studies.

Closely linked to the above, due to the limited number of participants required in SCEDs, the adoption of SCEDs could help address research design limitations within the rehabilitation literature by enabling the incorporation of *both* active and passive control groups in the same analysis in so doing, enabling the evaluation of the ‘active ingredient’ within the treatment arm ( Evans *et al.*, 2014; Krasny-Pacini & Evans, 2018). Additionally, due to SCED’s individualised and meta-cognitive nature, these designs have the added benefit of incorporating shorter intervention sessions (15 minutes), interspaced with longer sessions (30-45 mins), thereby allowing for regular follow-up of shorter periods (two weeks), juxtaposed with longer follow-ups (four to eight weeks) ( Evans *et al.*, 2014; Krasny-Pacini & Evans, 2018; Manolov & Moeyaert, 2016) to evaluate the efficacy of neuro-rehabilitation protocols.

The reviewed literature further indicates that a limited number of studies report biological marker data, such as patient viral loads, before and after cognitive rehabilitation intervention. To this end, previous studies ( *e.g.*, Benki-Nugent & Boivin, 2019) have found that (a) people living with HIV with CD4+ T-cell counts lower than 500 cells/μL are more likely to indicate HAND. Conversely, data suggests that (b) participants with lower viral loads and higher CD4+ T-cell counts may experience significant benefits from cognitive rehabilitation therapy ( Brahmbhatt *et al.*, 2017). It is therefore recommended that future studies conducting cognitive rehabilitation in the era of neuroHIV, report objective bio-marker data, such as viral load data, to complement neuropsychological measures indicating changes due to the cognitive rehabilitation. In line with the above observation, it is recommended that cognitive rehabilitation studies further supplement post-rehabilitation findings with objective brain imaging techniques such as functional near-infrared spectrometry (fNIRS), EEGs or other affordable neuroimaging markers to ascertain the efficacy of brain training protocols.

Lastly, several of the reviewed studies highlight the evolving nature of HIV/AIDS, especially the fact that the neuropsychological and neurobiological sequelae of HIV differ from population to population ( Brahmbhatt *et al.*, 2017; Brew & Garber, 2018). Consequently, it is recommended that cognitive interventions implement context-specific population norms, paired with specific cognitive rehabilitation protocols, supplemented with specific objective biomarker evaluations ( *e.g.*, fNIRS or CD4 viral load data). These context-specific norms could form the blueprint for cognitive rehabilitation studies regarding expected trajectories or outcomes related to the implementation of cognitive rehabilitation protocols within the specific context of interest, for example, in SSA or other low to middle-income settings with a heavy burden of neuroHIV.

## Data Availability

All data underlying the results are available as part of the article and no additional source data are required. Figshare: The cognitive remediation of attention in HIV Associated Cognitive Disorder: A Meta-Analysis and Systematic Review, https://doi.org/10.6084/m9.figshare.22196833 ( Zondo, 2023). This project contains the following extended data:
• Study Protocol.doc • Prisma Flow Diagram.pdf • Study Prisma Checklist.docx • Supplementary Materials.docx (Full Search Strategy, Relevant but Excluded Studies, Attention Outcome Measures, Study Quality Assessment) Data are available under the terms of the Creative Commons Attribution 4.0 International license (CC-BY 4.0).

## Footnotes

^1^
Within the extant literature, the terms ‘HAND’ and ‘neuroHIV’ are used concurrently to indicate cognitive impairment following HIV ( Saloner & Cysique, 2017).

^2^
CPE ranks all ARVs on four categories (a) physicochemical properties, (b) concentrations achieved in the CSF, (c) efficacy based on CSF virologic suppression, and (d) neurocognitive improvement ( Letendre *et al.*, 2008).

^3^
Within the extant literature, the terms ‘cognitive rehabilitation therapy’, ‘cognitive-training intervention’, and ‘brain training’, are used interchangeably ( Shawn Green *et al.*, 2019; Simons *et al.*, 2016), and this sequence will be equally applied in the manuscript.

^4^
Valid measures of attention were cross checked and validated based on ( Lezak *et al.*, 2004; Strauss *et al.*, 2006).

^5^
Following personal correspondence with multiple authors, the blinding of assessors to the experimental condition was not possible in most cases due to (a) resource constrains, (b) limited time frames to conduct research, and (c) the pilot nature of most studies in the field.
