# Supplementary material for: The cognitive remediation of attention in HIV-associated neurocognitive disorders (HAND): A meta-analysis and systematic review
Source: F1000Res. 2023 Sep 11;12:1133. [Version 1] doi: 10.12688/f1000research.132166.1 (PMC11109681; doi:10.12688/f1000research.132166.1)
Supplement: Peer review history

# Reviewer report: Reviewer response for version 1

**DOI:** 10.5256/f1000research.145072.r272773

**Published:** 2024-05-21

**Authors:** Antonio Lentoor

**Affiliations:** 1 Dept. of Clinical Psychology , School of Medicine, Sefako Makgatho Health Sciences University, Pretoria, Gauteng, South Africa

The article describes a systematic review and meta-analysis of published studies on an important topic relevant to the efficacy of cognitive rehabilitation intervention for attention remediation of HIV-associated cognitive disorders in the era of HAART.

 Similarities and contrasts between the selected studies were found via the narrative synthesis. The results of the meta-analysis demonstrate the effectiveness of cognitive rehabilitation as an intervention for improving attention in HIV-associated cognitive disorders (HANDS). In the context of HAART, this is pertinent and helpful for the study of HIV and brain health, particularly in Africa. There is currently little systematic clinical suggestion or consensus regarding the interventions most suitable for this population in the African environment. As this study fills a vacuum in rigorous research evaluating the effectiveness of cognitive rehabilitation intervention, it further emphasizes the significance of cognitive rehabilitation interventions for this population and environment. The results point to crucial areas for more study, especially in the African context.

 Generally, the study is conducted per the Cochrane Handbook guidelines, while the article is well-written in compliance with the standard reporting guidelines- PRISMA. The number of electronic database searches carried out was sufficient. Appropriate PICOS inclusion criteria were adhered to and in keeping with addressing the research aim.

 The results section answers the research question reliably and adequately. This is supported and presented in tables, and figures.  The author considered several important moderating factors in the subgroup analysis (duration, type, setting of the rehabilitation), study design, data quality, population, and type of study group to establish the effect size of interventions. The study also measured for heterogeneity (with a graphical forest plot illustration) of the intervention based on the *Q* statistic in the meta-analysis. A total of 14 studies were included for analysis. Quality assessments of these studies were also conducted, reflected in narrative text. In addition, a funnel plot with a symmetrical visual illustration is suggestive of minimal detected bias.

 I do not find any major concern in the article; I believe it is worth the approval. No further changes are required from my point of view.

Are the rationale for, and objectives of, the Systematic Review clearly stated?

Yes

Is the statistical analysis and its interpretation appropriate?

Yes

If this is a Living Systematic Review, is the ‘living’ method appropriate and is the search schedule clearly defined and justified? (‘Living Systematic Review’ or a variation of this term should be included in the title.)

Not applicable

Are sufficient details of the methods and analysis provided to allow replication by others?

Yes

Are the conclusions drawn adequately supported by the results presented in the review?

Yes

Reviewer Expertise:

NeuroHIV; Cognition; CNCD and Cognition; Neuropsychology

I confirm that I have read this submission and believe that I have an appropriate level of expertise to confirm that it is of an acceptable scientific standard.

# Reviewer report: Reviewer response for version 1

**DOI:** 10.5256/f1000research.145072.r272767

**Published:** 2024-05-10

**Authors:** David E Vance

**Affiliations:** 1 University of Alabama at Birmingham, Birmingham, USA

I appreciate the opportunity to review this well-written and thoughtful systematic review and meta-analysis on cognitive interventions to improve attention in people with HIV-associated neurocognitive disorder.  The rationale for, and objectives of, the systematic review and meta-analysis are clearly stated.  In the Introduction, the author clearly provided a compelling opening in the first paragraph and summarized the literature of how HIV impacts brain health leading to cognitive impairments in some individuals.  I do think it is important to state however that most people with HIV do not experience such cognitive problems; sometimes when reading literature like this, it is important to provide this balance as the lay population may just assume this occurs in everyone and catastrophizes the scope of the problem.  The author provides a succinct explanation of why non-pharmaceutical approaches to addressing cognitive impairment in people with HIV; this leads logically to the focus on cognitive rehabilitation (i.e., cognitive training) approaches as a burgeoning solution.

 Sufficient details of the methods and analysis are provided to allow replication by others.  This systemic review and meta-analysis provide the accepted step-by-step methodology in which the studies were chosen, evaluated, and data analyzed.  The cognitive rehabilitation studies had to examine some type of cognitive intervention (i.e., computer based, pencil and paper, hybrid) to determine if it was effective in producing meaningful change in attention scores in pre-post assessment.  Diligence was applied to the selection of articles.  Several search terms were used across databases (i.e., ERIC, Google Scholar, etc.) to locate potential articles that met eligibility criteria.  The eligibility criteria (i.e., inclusion criteria) for selecting the articles was logical as it addressed the purpose of the research question.  From this, 16 studies were included in the qualitative synthesis and 14 studies were included in the meta-analysis/quantitative analysis.  Standard PRISMA and PICO rubrics were applied.  It was not registered with PROSPERO, but it was registered on Protocols.io.  I was not familiar with Protocols.io but this appears to be a way to share one’s more detailed methodology and have it publicly available.  As I’ve worked with PROSPERO, it can very difficult and arcane to register studies and be rejected for unknown reasons; so, I am fine with this not being registered with PROSPERO as it is not always straightforward in knowing how or why it will take some registration of studies and not others as feedback is not provided.  But I digress.

 A unique and laudable aspect of this meta-analysis is that it considered several key moderators into the meta-analysis.  This is so desperately needed in the cognitive rehabilitation field already, but it was expertly applied in this article.  In Table 2, such moderators included duration of training (<10 sessions; 10 sessions; >10 sessions), type of samples (paediatric HIV vs geriatric HIV), type of control group, and so forth.  These moderation variables were all excellent, yet there might be some that are missing.  For example, instead of duration of training measured in sessions, I would find it better to include hours of training instead of number of sessions.  Yet, in the Discussion, this author argues, and I agree, that shorter sessions interspersed with longer sessions may be efficacious.  I would argue further that the length of sessions should be self-directed by the participant who can better monitor his/her engagement, fatigue, and interest level; and thus, self-directed training of variable time periods may be more optimal for therapeutic benefit.  Again, I digress. 

 Other moderator variables that I would have liked to see include in person lab training vs at home training.  I recognized that some of these studies might have required people to do the training in the lab while others were conducted at home or in community settings such as a library.  Location of training may provide different benefits.  In the lab setting, there is certainly less distraction from extraneous noise and daily interruptions which may allow one to focus more on the cognitive rehabilitation tasks, yet this is an artificial environment.  Likewise, in the lab setting, one can usually reach out to research staff who can easily trouble shoot any technical difficulties that arise, especially with the computerized cognitive rehabilitation approaches.  Yet, at home training may provide more flexibility and is a more ecologically valid place in which we as cognitive researchers want the rehabilitation to occur, that is to be put in the community and in the hands of those who need and would benefit from the intervention.  It is not clear if these studies were conducted in the lab setting or if some were done in the community or more preferably for participants, at home.  After the COVID-19 pandemic, globally we have grown accustomed to doing more things in the comfort of our homes as technology has expanded to accommodate this new reality; examining such cognitive interventions in the home environment would be a logical next step.  Even if the studies in this review were not occurring in a home setting, providing such a discussion is warranted. 

 There are three areas that I’m less clear after reading the methods, and that concerns: 1) HAND diagnosis, 2) type of attention training, and 3) type of attention performance measure.  First, concerning HAND, the title states that these studies look at cognitive rehabilitation in those with HAND.  There are a variety of methods to measure HAND, and studies have shown that different types yield different prevalence rates of HAND in the same sample.  There is not much we can do about that, but in Table 1, or elsewhere, it may be helpful to say how HAND was measured in each of the studies as these obviously vary from study to study.  Second, concerning type of attention training, I was uncertain if it was being argued that the cognitive rehabilitation studies were using interventions specifically designed to improve attention or were the studies targeting a specific cognitive ability (i.e., speed of processing) but the researchers happened to measure attention before and after the training that may or may not have been focused on improving attention specifically.  I think it is fine either way, but perhaps it should be stated in Table 1 or elsewhere (supplemental file) what was being done in each study.  And finally, it is not clear how attention is being measured in each study; it would be helpful to name the specific cognitive performance test for each study in which attention is measured.  As neuropsychologists, sometimes we agree and often disagree on what we consider a specific cognitive performance test is measuring.  I will not dive into splitting those hairs, but again, I think it should be reported in Table 1 or elsewhere.

 The statistical analysis and its interpretation are appropriate.  These are typical approaches for conducting meta-analyses (i.e., forest plot, mean differences, funnel plot).  With the funnel plot, I’ve not seen this done and I don’t do this with my meta-analytic studies but I might, but perhaps we can identify the dots to the particular study (although not necessary, just a thought).  The subgroup analyses were certainly an added bonus of this meta-analysis; something I don’t see often.  Kudos!

 The conclusions drawn are adequately supported by the results.  There were no surprises; this made sense.  I agree with the comments that braining training interventions can be fatiguing, time consuming, and prone to high attrition rates.  As such, it does make me question whether attrition rates and completion rates of the intervention should be considered in Table 2 as one of the moderators.  Otherwise, I whole heartedly agree that more research should be invested in this approach especially concerning examining its mechanistic properties using brain imaging; I would also suggest examining whether it changes neurochemical levels (i.e., BDNF, NfL) in the brain and periphery.

 Overall, this article is a welcome addition to the scientific literature on cognitive rehabilitation in neuroHIV.  This article provides new methodological insights and considerations for future research.

Are the rationale for, and objectives of, the Systematic Review clearly stated?

Yes

Is the statistical analysis and its interpretation appropriate?

Yes

If this is a Living Systematic Review, is the ‘living’ method appropriate and is the search schedule clearly defined and justified? (‘Living Systematic Review’ or a variation of this term should be included in the title.)

Partly

Are sufficient details of the methods and analysis provided to allow replication by others?

Yes

Are the conclusions drawn adequately supported by the results presented in the review?

Yes

Reviewer Expertise:

I am a psychologist who studies cognitive aging with HIV and conduct cognitive interventions in those with HIV.

I confirm that I have read this submission and believe that I have an appropriate level of expertise to confirm that it is of an acceptable scientific standard.
